# Editorial: Temporal Structure of Neural Processes Coupling Sensory, Motor and Cognitive Functions of the Brain

**DOI:** 10.3389/fncom.2020.00073

**Published:** 2020-09-15

**Authors:** Daya Shankar Gupta, Arpan Banerjee, Dipanjan Roy, Federica Piras

**Affiliations:** ^1^Biology Department, Camden County College, Blackwood, NJ, United States; ^2^Cognitive Brain Dynamics Lab, National Brain Research Center, Gurugram, India; ^3^Laboratory of Neuropsychiatry, Department of Clinical and Behavioral Neurology, IRCCS Santa Lucia Foundation, Rome, Italy

**Keywords:** binding, perception, multi-scale timing, temporal coupling, mutual information, time-dimension in the brain

## Introduction

Temporal structure of cognitive and sensory processing holds the key to understanding complex neural mechanisms involved in higher order brain functions like perception of time. A hypothesis of embodied cognition posits that cognitive processes are deeply rooted in the interactions with the external world (Wilson et al., 2002; Anderson et al., 2012). These interactions of the brain with the external world depend on the accurate representation of the time-dimension in neural circuits (Gupta, 2014). For example, one cannot catch a flying ball unless the timing of the movements matches the speed of the ball. Many real world situations depend on the mapping between the neural and physical representation of time, which is maintained at different hierarchical levels. Hierarchical processing, consistent with multiple time scales, is manifested during goal-driven tasks, such as interval timing, duration judgement, and movement coordination. Contributions to this Research Topic elucidate how key aspects of the time-dimension such as the temporal binding of neural events play important roles in various cognitive processes, which include perception, mental time travel, and speech production. Additionally, the multi-scale representation of such processes from the micro to meso scales—from single neurons to a population of neurons to field potentials and macroscopic scales of EEG - is, discussed.

## Contributions

Hashimoto and Yotsumoto studied an oscillator-based model of time perception by recording EEG data during interval timing tasks. They observed that the duration reproduction of a visual stimulus, flickering at 10 Hz, was 1.22 times longer than constantly illuminated stimulus. The EEG data further revealed an event-related potential (ERP), phase-locked to the flicker, fluctuating at 10 Hz, suggesting an increase in the certainty about the physical time-dimension in the neural circuits of the brain, which can be analyzed as mutual information. Authors also found desynchronization of spontaneous neural oscillations during the flicker observation period, which is consistent with the presence of desynchronization during information processing (Kumar et al., 2020). Furthermore, during time reproduction, there was an increase of the spontaneous alpha oscillation amplitude. This is consistent with the synchronization of distributed oscillators by neural oscillations in the calibration of local circuits during time reproduction tasks (Gupta, 2014). Thus, the phase-locked 10 Hz ERP induced by the flickering stimulus is likely to improve the accuracy of neural timing mechanisms in the brain.

Arguably, an increase in the error in timing tasks will lead to an underestimation of time intervals. This is based on the likely effect of evolutionary pressures, whenever there is less certainty about the physical time-dimension in neural circuits we determine a decrease in interval timing accuracy. Indeed, the underestimation of time intervals, when accuracy of timing mechanisms decreases due to the allocation of neuronal resources/attention to other environmental aspects, will confer a survival advantage, for example, protecting oneself from an incoming projectile or catching fruit dropping at a certain speed from the tree. The same yet to be determined mechanism, which results in the underestimation of time due to a decrease in accuracy, could cause time-interval overestimation if there is an improvement in accuracy. Thus, the stimulus-induced 10 Hz neural entrainment, which increases the certainty of the time-dimension in neural circuits, would result in the overestimation of the time interval.

Ben-Soussan and Glicksohn studied the effect of 1 month of Quadrato Motor Training (QMT), a type of motor training, on time production in dyslexic patients. In contrast to the control subjects, the dyslexic subjects produced longer intervals. Authors argue that longer time interval production is due to increased attention. QMT activates many parts of the brain simultaneously, such as areas that are responsible for motor control and language-related functions. Thus, QMT training will result in an increased ability to simultaneously keep many networks active, which may increase neuronal resources for attention. Development of additional neuronal resources could also improve the calibration of the clock mechanisms (Gupta, 2014). This would increase accuracy of internal neural clocks, which as hypothesized, could be responsible for the over-production of time intervals.

The magnitude of time-intervals can also affect perception. This is suggested by the findings of Wan and Chen, who showed using the Ternus display in a forced choice task that prior exposure to longer mean (or last) auditory intervals elicited more reports of group motion, whereas the shorter mean (or last) auditory interval gave rise to more dominant perception of element motion. Although longer intervals, greater than 50 ms, promoted group motion in general, the longer auditory intervals, which are also a more efficient form of the time-dimension input (Comstock et al.), can produce their effect by increasing the certainty of longer time intervals in the neural circuits processing the Ternus display, determining a greater report of group motion. Likewise, the exposure to shorter auditory intervals prior to the forced choice task, which would increase the certainty of shorter time intervals, results in a greater report of element motion. The role of increased certainty is consistent with theory by Gupta and Bahmer (2019), who proposed that perception is the outcome of an increase in mutual information, as well as surprisal.

Slater and Tate highlight the significant overlap between neural systems involved in processing rhythm and those implicated in Attention Deficit Hyperactivity Disorder (ADHD). Authors link the impaired attentional control seen in ADHD to their rhythm-related deficits. They assert that the same neural bases—from the brain circuitry to dopamine signaling—that support the processing of musical rhythm are implicated in ADHD. Additionally, they present computational models of rhythm perception, based on the entrainment of multiple neural oscillators (Large and Palmer, 2002; Slater and Tate). The multiple-oscillators model can also provide a basis to represent the time-dimension, as well as the transfer of timing information from one modality to another in the brain (Gupta, 2014), for example, from auditory to visual, as suggested by the cross-modal interaction reported by Wan and Chen.

Ravignani et al. presented a mathematical model to explore small integer-ratio bias in rhythm perception and production. This small integer bias is likely to be also reflected in the representation of the time-dimension in neural oscillators, which would be responsible for the input of time-intervals that are small integer ratios. This could explain better perception for integer-ratio stimuli over more complex metrical patterns (Large and Kolen, 1994), which is in line with the role of temporal duration in perceptual functions as mentioned above (Wan and Chen).

In subjects doing mental time travel tasks, Schurr et al. recorded via electrodes inserted in the hippocampus and the lateral temporal cortex (LTC). Recordings revealed early modulatory activity between ~100 and 300 ms in the left LTC, followed by later activity in the left hippocampus between ~400 and 600 ms, which were independent, as shown by electrode classification. The authors suggest that this represents a division of labor. Additionally, separation by about 100 ms between two modulations, that are not correlated, also suggests that the activities in the left LTC and the hippocampus are temporally coupled, which could serve as a basis for the overall experience of mental time travel. It should be noted that, in addition to a synchronous occurrence, two or more neuronal events may be temporally coupled by a non-zero duration separating them. Moreover, many temporally coupled neural events may not be correlated, unless they are also causally related, for example, by an external stimulus or brain oscillation (Gupta and Bahmer, 2019). As suggested by this study, temporal coupling of neural events, without correlations, could contribute to brain cognitive functions, including the perception of time durations of various scales.

In a review article, Bahmer and Gupta have argued that temporal coupling, especially coincidental activation of neural circuits, is responsible for perceptual functions of the brain. Simulations showed that few chopper neurons, employing coincidental activation, can generate different inter-stimulus intervals, which can contribute to pitch perception at the cortical level, especially in a difference of 0.2% in pitch by sensitive listeners.

Pouget et al. have shown using a task of voluntary breathing control, that intentional initiation of breathing occurred when premotor EEG recorded potential reached a threshold. Furthermore, the reaction time in the voluntary initiation of breathing was correlated to the amplitude of the threshold, which varied stochastically. Hence, the initiation of breathing occurred according to a stochastically distributed reaction time. The stochastic distribution of breathing initiation times is consistent with the role of surprisal information in information processing during speech production (Gupta and Bahmer, 2019). Gupta and Bahmer (2019) have argued that surprisal information combined with the increase in mutual information plays an important role in the mental representation of perceptual objects, such as the specific contents of speech.

Wang et al. have shown that English alphabet letters (vowels and the letter t) can be decoded from phase and power of EEG oscillations over the occipital and temporal regions. However, in comparison to the phase, the power of oscillation was less effective in decoding. It should be noted that an increase in oscillation power indicates that more oscillations are in the same phase, which would reduce the effectiveness of multiple individual spikes in representing information. In this study, synchronization appears, at least to some extent, to represent information about the alphabets.

Soman et al. have proposed an autoencoder model based on an oscillatory neural network, which is tested using real life situations and synthetic EEG signals. Autoencoders encode signals, perform dimensionality reduction, and decode the signals. As the authors point out, in the human brain dimensionality reduction occurs when ~125 million photoreceptors converge to ~1 million neurons in the lateral geniculate nucleus and then the visual information spreads to the primary visual areas. The proposed autoencoder uses biologically plausible features of neuronal oscillatory systems, such as phase synchronization, frequency tuning, and convergence. For example, convergence of inputs in the encoder, combined with an inhibitory network, maximizes the variance, which extracts useful features of the input, while resulting in dimensionality reduction. The study of autoencoders as a computational model can shed light on the functions of various relay centers throughout the nervous system. Moreover, in the relay centers, autoencoder-like functionality, such as synchronization, can incorporate time-dimension into information processing.

Lloyd et al. adapted network analysis from graph theory to reveal structures in time (rather than in space) in fMRI image series in healthy subjects at rest, or passively viewing a movie, from the human connectome project. In their analysis, each whole brain image is a temporal node, i.e., a “moment.”. Collections of correlated moments or nodes across a time-interval, comprising time points where patterns of global brain activity are similar—referred to as themes—were significantly detected. The authors also found rhythms and harmonies in the patterns of themes, which were broadly similar in two different experimental conditions. They hypothesized that the detected rhythms and their harmonic relationship suggests that harmonic signaling might be adaptive from a computational point of view. Further analysis of themes revealed that sequences of 6 or 7 s were most often rhythmic. Rhythmic sequences of 6 to 7 s length are unlikely to play a direct role in the information processing underlying direct perception and action. However, it would be interesting to see if these rhythmic sequences play a role in higher mental functions, such as planning and mental time travel.

Gili et al. argue that metastable dynamics underlie the interactions between parts of the brain necessary for its dynamic functioning associated to time perception. Metastability can be understood as an energy landscape for an ensemble of possible states, which define the phase space of the brain system. These possible states tend to achieve local and global minima. Unlike synchronization, which constrains synchronized parts of the brain, metastable states are characterized by a tendency for interacting parts of the brain to be independent. The authors argue that metastable states underlie time perception in multimodal sensory processing when different parts of brain may be independently processing different sensory modalities or serving motor functions.

Yang et al. studied how wrist stretch (perturbation) modulated the effective connectivity for the early and late periods between multiple brain areas, which included the primary somatosensory cortex, primary motor cortex, premotor cortex, supplementary motor area, and the posterior parietal cortex on both hemispheres. Dynamic causal modeling was applied to analyze the connectivity between different areas and its modulations when a constant torque was applied in the presence of the external perturbation. There were greater modulations in the late phase 100–350 ms post-perturbation compared to 20–100 ms post-perturbation. This work highlighted interactions between motor and sensory areas during movements, which would reflect the interaction of the brain with the four-dimensional physical world. An increase in connectivity, which is an estimate of mutual information, would play an important role in the temporal processing of information underlying the perceptual functions of the brain.

Das and Ray used spike-LFP coherence to test if the phase coding by gamma rhythm varies with stimuli contrast or attention in V1. Here the authors use phase coding (PC) to test a specific hypothesis: whether stimuli contrast or attentional load can change the position of the spike relative to the phase of gamma frequencies in LFP. To be phase coded, spikes resulting in stronger activation of pyramidal cells appear earlier in gamma cycles as they overcome the inhibition of pyramidal cells earlier. Interestingly, they report only a weak effect of attention on the spike-field phase relationship of PC in V1, which contrasts with the findings of Fries et al. (2007) and Fries (2015). On the macroscopic scale, however, Peng et al. argue that visual crowding, which is mainly attributed to processing in early visual areas, can be modulated by top-down attention.

## Conclusion

In our endeavor to understand time-dimension as a bridge to integrate multi-scale observations of behavior and brain information processing, the contributions in this Research Topic have revealed certain key aspects of time-dimension. These include the possible role of temporal coupling by non-zero intervals and the effects of an increase in mutual information in neural circuits on perception and cognitive functions, given different aspects of physical time-dimension. Important future goals at this juncture should include (a) a study of temporal coupling between unrelated neural events and (b) an increase in mutual information in the brain, given the temporal characteristics of external events, such as speed and rhythmicity.

## Author Contributions

All authors listed have made a substantial, direct and intellectual contribution to the work, and approved it for publication.

## Conflict of Interest

The authors declare that the research was conducted in the absence of any commercial or financial relationships that could be construed as a potential conflict of interest.
